# Colocolic Intussusception in Children: A Case Series and Review of the Literature

**DOI:** 10.3389/fsurg.2022.873624

**Published:** 2022-04-06

**Authors:** Jiayu Yan, Qiulong Shen, Chunhui Peng, Wenbo Pang, Yajun Chen

**Affiliations:** ^1^Department of General Surgery, National Center for Children's Health, Beijing Children's Hospital, Capital Medical University, Beijing, China; ^2^Department of Emergency Surgery, National Center for Children's Health, Beijing Children's Hospital, Capital Medical University, Beijing, China

**Keywords:** colocolic intussusception, children, pathologic lead point, treatment, open surgery

## Abstract

**Background:**

Colocolic intussusception is a rare subtype of intussusception mostly caused by juvenile polyps. The treatment of colocolic intussusception caused by other pathologic lead points in children remains poorly understood.

**Method:**

A systematic literature review was performed between January 2000 and June 2021 to characterize the comprehensive treatment of colocolic intussusception in children. This report also included patients admitted to our center between January 2010 and June 2021 who were not previously reported in the literature.

**Results:**

We identified 27 patients in 20 studies in addition to 17 patients from our center for a total of 44 patients (median age, 4.4 years; 52.3% male). The lead point was identified in 40 patients (40/44, 90.9%). The most common lead point was juvenile polyps (19/44, 43.2%). A therapeutic enema was performed in 15 patients with colocolic intussusception caused by juvenile polyps and was successful in 9 patients (9/15, 60.0%). Colonoscopic polypectomy was subsequently performed in 8 patients and was successful in 7 patients (7/8, 87.5%). The other patient had undergone laparoscopic exploration, and no abnormality was found. Subsequently, the patient underwent open surgery. The patients with colocolic intussusception caused by other pathologic lead points almost underwent surgical treatment (15/21, 71.4%), including 13 open surgeries and 2 laparoscopic surgeries.

**Conclusion:**

A therapeutic enema followed by colonoscopic polypectomy is feasible to treat colocolic intussusception caused by juvenile polyps unless the patient has bowel perforation; however, surgery is sometimes needed. For patients with colocolic intussusception caused by other pathologic lead points, open surgery remains the primary treatment.

## Introduction

Intussusception is one of the most common abdominal emergencies in children. It often occurs near the ileocecal junction and rarely only involves the colon (1, 2). Approximately 90% of ileocolic intussusception cases are idiopathic, and most can be resolved by nonoperative reduction with therapeutic enema (2, 3). However, almost all colocolic intussusception is caused by pathologic lead points and should be treated with surgical interventions, including colonoscopic polypectomy, open surgery or laparoscopic surgery (4–8). The most common lead point in colocolic intussusception is juvenile polyps (6).

The available literature on colocolic intussusception is sparse, and consists mainly of case reports. Some case reports have shown that a successful therapeutic enema followed by colonoscopic polypectomy is feasible for treating colocolic intussusception caused by a juvenile polyp, but open surgery is still used in most cases (6, 7, 9). Additionally, the effect of laparoscopic surgery on colocolic intussusception in children is controversial, and is often used for surgical exploration, and the treatment of colocolic intussusception caused by other lead points remains poorly understood. Thus, we performed this retrospective study to summarize the clinical features of colocolic intussusception using the largest sample size in China and a review of the literature to provide evidence that could be used to improve treatment in clinical practice (10).

## Materials and Methods

### Study Population

After approval by the Ethics Committee of Beijing Children's Hospital (approval number [2021]-E-090-R), the medical records of all children diagnosed with intussusception and admitted to Beijing Children's Hospital between January 2010 and June 2021 were retrospectively reviewed. Informed consent was waived due to the nature of the study. Patients diagnosed with colocolic intussusception and confirmed by ultrasound, colonoscopy or surgery were included. The collected data included demographics, clinical symptoms, accompanying malformations, imaging data, clinical interventions, and pathological data.

### Search Strategy

The study was performed in accordance with the PRISMA guidelines. The following databases were searched through June 30, 2021: PubMed, Web of Science and the Cochrane Library. Search strings included colocolic intussusception, pediatric, child and children (Table 1). The reference lists of the relevant studies were manually searched to identify other potentially appropriate studies. Retrospective studies, including case reports and case series that assessed the treatment of colocolic intussusception, were included. Studies investigating adults, reviews, studies not written in English, studies lacking complete data, and studies without the outcomes of interest were excluded. Additionally, to explore the progress of the treatment of colon intussusception and provide evidence that would be useful in clinical practice, studies published before 2000 were also excluded.

**Table 1:** Database search results.

**Search**	**Colonic**	**Colonic**	**Colonic**
**term**	**intussusception**,	**intussusception**,	**intussusception**,
	**Pediatric**	**Child**	**Children**
PubMed^a^	11	25	6
Web of Science^b^	9	19	19
The Cochrane Library^c^	2	3	3

a *Search query: (**colonic** intussusception [Title/Abstract] AND pediatric [Title/Abstract]), (**colonic** intussusception [Title/Abstract] AND child [Title/Abstract]), (**colonic** intussusception [Title/Abstract] AND children [Title/Abstract])*.

b *Search query: TS, (**colonic** intussusception AND pediatric); TS, (**colonic** intussusception AND child); TS, (**colonic** intussusception AND children)*.

c *Search query: colonic intussusception in Title Abstract Keyword AND pediatric in Title Abstract Keyword, colonic intussusception in Title Abstract Keyword AND child in Title Abstract Keyword, colonic intussusception in Title Abstract Keyword AND children in Title Abstract Keyword*.

### Data Analysis

Categorical variables are presented as numbers and frequencies. Continuous variables with a normal distribution are presented as the means ± standard deviations (SDs), and those with a nonnormal distribution are presented as the medians and IQR. The statistical calculations were performed using a software program (IBM SPSS Package, version 22; IBM Corporation).

## Results

### Authors' Cases

A total of 1,322 patients were diagnosed with intussusception and admitted to Beijing Children's Hospital between January 2010 and June 2021. Among these patients, 17 (1.3%) were diagnosed with colocolic intussusception (Table 2). The median age at presentation was 6.4 ± 4.9 years, and 6 patients (6/17, 35.3%) were male. Seven patients (7/17, 41.2%) had been misdiagnosed at other hospitals. Abdominal ultrasound was performed in all patients, with a positive rate of 100.0% to diagnose colocolic intussusception. Juvenile polyps were identified as the lead point in 11 patients (11/17, 64.7%). Therapeutic air enema was performed in 9 patients diagnosed with colonic polyps by ultrasound, which successfully reduced intussusception in 6 patients. Among the patients in whom therapeutic air enema was successful, 5 with pedunculated polyps underwent colonoscopic polypectomy, and the other patient, who had a sessile polyp, underwent segmental colonic resection. Colon duplication was identified as the lead point on ultrasound in 2 patients, and surgical reduction with segmental colonic resection was subsequently performed. Multiple polyposis was identified as the lead point on ultrasound in 3 patients. Considering the severe abdominal pain and signs of peritonitis, the patient with familial adenomatous polyposis underwent an emergency ileocecal resection and enterostomy after unsuccessful therapeutic air enema. The patient was waiting for radical surgery. The other 2 patients underwent colonoscopic polypectomy to confirm the diagnosis as adenocarcinoma caused by adenomatous polyps and Peutz-Jeghers polyp, respectively. The patient with adenocarcinoma further underwent segmental colonic resection combined with postoperative adjuvant chemotherapy at another hospital. The patient with Peutz-Jeghers syndrome underwent another colonoscopic polypectomy for abdominal pain 3 months later. At follow-up, the above 2 patients did not present relevant symptoms. The last patient was diagnosed with colocolic intussusception caused by hemangioma, but no further treatment was performed after successful therapeutic air enema.

**Table 2:** Clinical features of 17 patients with colocolic intussusception at our center between 2010 and 2021.

**Case**	**Gender**	**Age (years)**	**Symptoms**	**Misdiagnosis**	**Ultrasound**	**Air enema**	**Treatment**	**Location/lead point**
1	Female	6.9	Abdominal pain, palpable mass, bloody stool	Enterospasm	Colocolic intussusception (+) Polyp (+)	Yes, successful	Colonoscopic polypectomy	Descending colon Juvenile polyp
2	Female	5.3	Abdominal pain, palpable mass	No	Colocolic intussusception (+) Polyp (+)	Yes, successful	Colonoscopic polypectomy	Descending colon Juvenile polyp
3	Male	2.6	Abdominal pain, palpable mass, bloody stool	Gastroenteritis	Colocolic intussusception (+) Polyp (+)	Yes, successful	Colonoscopic polypectomy	Descending colon Juvenile polyp
4	Male	3.3	Abdominal pain, bloody stool	Gastroenteritis	Colocolic intussusception (+) Polyp (+)	Yes, successful	Colonoscopic polypectomy	Splenic flexure Juvenile polyp
5	Female	6.3	Bloody stool	No	Colocolic intussusception (+) Polyp (+)	Yes, successful	Colonoscopic polypectomy	Transverse colon Juvenile polyp
6	Female	0.7	Abdominal pain, palpable mass, bloody stool	No	Colocolic intussusception (+) Polyp (+)	Yes, successful	Colonoscopy, open surgery	Descending colon Juvenile polyp
7	Male	8.2	Abdominal pain, vomiting	Enterospasm	Colocolic intussusception (+) Polyp (+)	Yes, unsuccessful	Colonoscopic polypectomy	Transverse colon Juvenile polyp
8	Male	7.1	Abdominal pain	No	Colocolic intussusception (+) Polyp (+)	Yes, unsuccessful	Colonoscopic polypectomy	Ascending colon Juvenile polyp
9	Female	3.2	Abdominal pain, palpable mass	Mesentery lymphadenites	Colocolic intussusception (+) Polyp (+)	Yes, unsuccessful	Open surgery	Descending colon Juvenile polyp
10	Male	2.8	Abdominal pain, diarrhea	Mesentery lymphadenites	Colocolic intussusception (+) Polyp (+)	No	Open surgery	Hepatic flexure Juvenile polyp
11	Female	4.2	Abdominal pain	No	Colocolic intussusception (+) Polyp (+)	No	Open surgery	Hepatic flexure Juvenile polyp
12	Female	3.2	Abdominal pain, vomiting	Mesentery lymphadenites	Colocolic intussusception (+) Enteric duplication (+)	No	Open surgery	Ascending colonic duplication
13^a^	Female	16.5	Abdominal pain, prolapsed bowel, bloody stool	No	Colocolic intussusception (+) Enteric duplication (+)	No	Open surgery, Colonoscopy	Total colonic duplication
14	Female	10.5	Abdominal pain, prolapsed bowel, bloody stool	No	Colocolic intussusception (+) Multiple polyp (+)	Yes, unsuccessful	Open surgery	Total colon Familial adenomatous polyposis
15^b^	Female	16.6	Abdominal pain	No	Colocolic intussusception (+) Multiple polyp (+)	No	Colonoscopic polypectomy	Descending colon Adenomatous polyp, Hepatic flexure Adenocarcinoma
16^c^	Female	11.8	Abdominal pain, vomiting	No	Colocolic intussusception (+) Multiple polyp (+)	No	Colonoscopic polypectomy, enteroscopy	Total colon Peutz-Jeghers polyp
17^d^	Male	0.1	Bloody stool	No	Colocolic intussusception (+) Hemangioma (+)	Yes, successful	——	Ascending colon Hemangioma

a *The patient underwent resection of the duplicated colon distal to the ascending colon and a side-to-end colon anastomosis due to total colon duplication at 2 months*.

b *The patient was diagnosed with acute lymphoblastic leukemia at 7 years old and the treatment was completed at the age of 10*.

c *The patient was diagnosed with Peutz-Jeghers syndrome at age 1*.

d *The patient was diagnosed with colocolic intussusception caused by hemangioma at ascending colon, but no further treatment was performed after successful therapeutic air enema*.

### Literature Search

**Figure 1:**
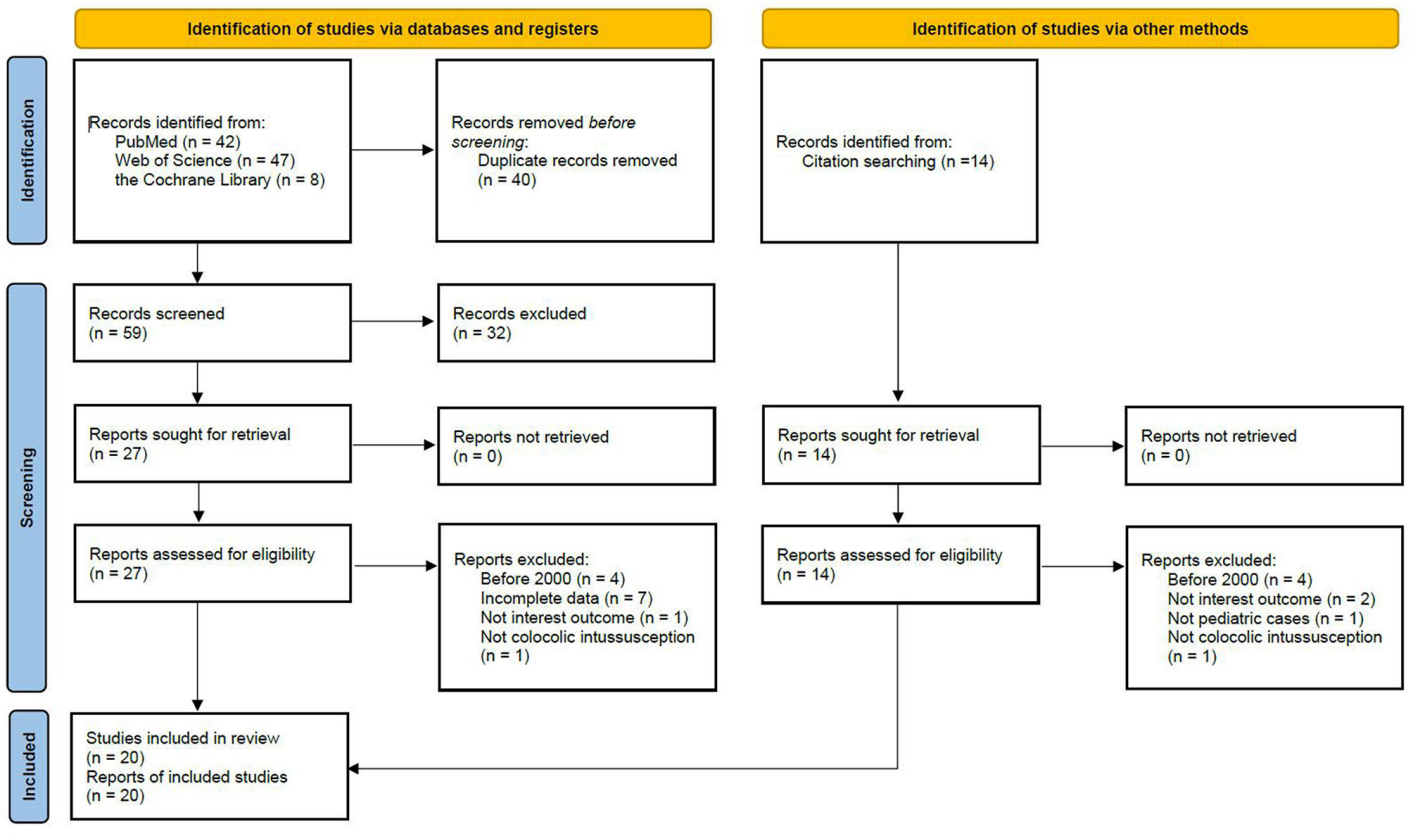
PRISMA flowchart for study selection.

The search identified 111 studies, including 97 from databases and 14 from citation searching (Figure 1). Forty-one studies had undergone full-text review, and 21 were excluded because they were published before 2000, lacked incomplete data, did not report the outcome of interest, and were not about colocolic intussusception or pediatric cases. Twenty studies were included, and 44 patients, including our 17 patients, were evaluated (Table 3) (5–24).

**Table 3:** Studies from 2000 in order of year of publication (*n* = 20).

**Year**	**Author (s)**	**Patients (n)**	**Age (years)**	**Gender**	**Location**	**Lead point**
2004	Pritzker et al.	1	11.0	Female	Left colon	Hereditary angioneurotic edema
2005	Abantanga et al.	1	3.0	Male	Terminal ileum	Ileal invagination of the sigmoid colon
2005	Hafen et al.	1	17.0	Male	–	No lead point
2008	Tennant et al.	1	0.2	Male	–	No lead point
2008	Soccorso et al.	1	5.0	Female	Sigmoid colon	Ganglioneuroma
2008	Rahim et al.	1	7.0	Male	–	No lead point
2009	Al-Jahdali et al.	1	0.2	Female	Unknown	Lymphangioma
2010	Utsumi et al.	1	2.8	Male	Cecum	Capillary hemangioma
2012^a^	Sanchez et al.	1	4.0	Female	Cecum	Submucosal edema with enlarged lymphatics
2012	Abrahams et al.	1	4.0	Male	Transverse colon	Juvenile polyp
2013	Das et al.	1	13.0	Male	Descending colon	Adenocarcinoma
2013	Simmi et al.	1	0.2	Female	Sigmoid colon	Gastrointestinal stromal tumor
2014	Takahashi et al.	1	10.0	Female	Transverse colon	Juvenile polyp
2014	Kurian et al.	1	0.3	Male	Cecum	Kaposiform hemangioendothelioma
2016	Tripathy et al.	1	7.0	Male	Transverse colon	Juvenile polyp
2016	Adorisio et al.	1	8.0	Male	Unknown	Enterobius vermicularis
2017	Eid et al.	1	10.0	Male	Left colon	Synovial sarcoma
2018	Rehan et al.	1	0.2	Female	Unknown	Infantile hemangioma
2018	Brundler et al.	1	1.5	Male	Transverse colon	Lipoblastoma
2020	Richer et al.	8	1.8	Male	–	No lead point
			2.6	Male	Left colon	Juvenile polyp
			3.5	Female	Left colon	Juvenile polyp
			4.0	Male	Left colon	Juvenile polyp
			5.5	Male	Left colon	Juvenile polyp
			14.6	Male	Left colon	Juvenile polyp
			4.6	Female	Left colon	Hamartomatous polyp
			7.8	Female	Left colon	Leiomyoma

a *Pneumatosis intestinalis by using steroids after heart transplant resulting in intussusception*.

### Characteristics, Diagnosis and Treatment

The median age at presentation was 4.4 (2.7, 8.2) years old. Twenty-three patients (23/44, 52.3%) were male. The lead point was identified in 40 patients (40/44, 90.9%). The most common lead point was juvenile polyps (19/44, 43.2%), followed by colon duplication, adenocarcinoma and hemangioma (2/44, 4.5%), and other lead points were rare and sporadic.

The diagnosis and treatment of patients are summarized in Table 4. The most common clinical symptoms were abdominal pain, bloody stool, and vomiting, which were identified in 88.6, 63.6, and 45.5% of the patients, respectively. Sixteen patients (16/44, 36.4%) had all three symptoms. Fifteen patients (15/44, 34.1%) had been previously misdiagnosed. Ultrasound was performed in 37 patients (37/44, 84.1%), 34 (34/37, 91.9%) with indications of intussusception. Abdominal X-ray was performed in 18 patients (18/44, 40.9%), 16 (16/18, 88.9%) with indications of intestinal obstruction. Computed tomography was performed in 9 patients (9/44, 20.5%), all of whom had indications of intussusception. Barium enema was performed in 5 patients (5/44, 11.4%), all of whom had indications of filling defects in the colon or rectum.

**Table 4:** Diagnosis and treatment of patients with colocolic intussusception (*N* = 44).

**Characteristics**	**Results^c^**
Age at presentation, years	4.4 (2.7, 8.2)
Symptoms^a^, *n* (%)
Abdominal pain	39 (88.6)
Bloody stool	28 (63.6)
Vomiting	20 (45.5)
Palpable mass	15 (34.1)
Diarrhea	9 (20.5)
Rectal mass or prolapse	7 (15.9)
Misdiagnosis^b^, *n* (%)	15 (34.1)
Diagnostic method, *n* (%)
Ultrasound	37 (84.1)
Abdominal X-ray	18 (40.9)
Computed tomography	9 (20.5)
Barium enema	5 (11.4)
Therapeutic enema (air/saline/ barium), *n* (%)	26 (59.1)
Treatment for intussusception, *n* (%)
Open surgery	18 (40.9)
Colonoscopy	15 (34.1)
Colonoscopy + Open surgery	3 (6.8)
Laparoscopic surgery	2 (4.5)
Laparoscopic surgery + Open surgery	1 (2.3)
Open surgery + Colonoscopy + Open surgery	1 (2.3)

a *Sixteen patients (16/44, 36.4%) had the three symptoms: abdominal pain, bloody stool, and vomiting*.

b *Misdiagnosis: 4 mesentery lymphadenitis, 4 infectious gastroenteritis, 2 fecal impaction, 1 Meckel's diverticulum, 1 Entamobea histolytica cysts, 2 enterospasm, 1 unknown*.

c *The above-summarized results were all mentioned in the included study and the characteristics that were not mentioned in the included study were considered to be absent*.

**Figure 2:**
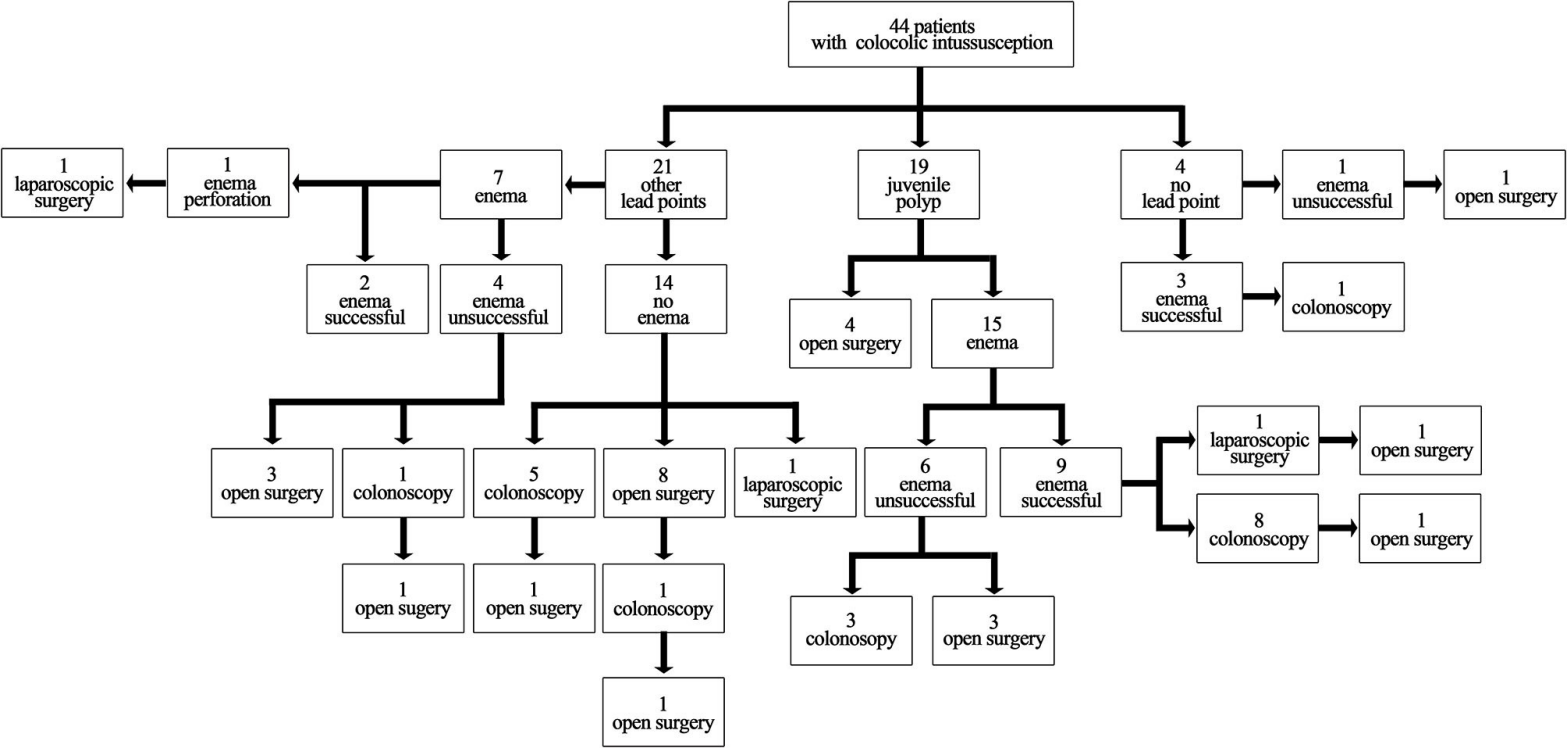
Details of the treatment for patients with colocolic intussusception (*N* = 44).

Details of the treatment administered to each patient are shown in Figure 2. Therapeutic enema was performed in 26 patients (26/44, 59.1%) and successfully reduced intussusception in 14 patients (14/26, 53.8%). Eighteen patients (18/44, 40.9%) underwent open surgery directly. Eighteen patients (18/44, 40.9%) underwent colonoscopy, among whom 3 switched to open surgery-1 with a sessile juvenile polyp, 1 with multilobulated broad-based lipoblastoma, and 1 with obstructive synovial sarcoma.

## Discussion

Colocolic intussusception in children is far less common than ileocolic intussusception, occurring in <5.0% of most case series except on the African continent, and most have a pre-existing colonic pathology acting as a lead point (5, 6, 25, 26). The incidence of colocolic intussusception was 1.3% at our center, a value lower than that reported in the literature. The reason might be related to ethnic and geographic differences. Additionally, patients with intussusception treated successfully by air enema at the outpatient department were not included in our case series, which affected the incidence. A review of the studies published in the past 20 years showed that unlike the male predominance reported in previous studies on all types of intussusception, no such predominance was identified among patients with colocolic intussusception, as reported in previous surveys (26). Our study also confirmed that the age at the development of colocolic intussusception was older on average than that of patients with ileocolic intussusception (27, 28). The reason might be associated with pathologic lead points, such as juvenile polyps, which occur most commonly in children older than 2 years of age (29).

The clinical symptoms of abdominal pain, bloody stool and vomiting are often considered the main features of intussusception. However, <25% of patients have this classic triad, leading to a delayed diagnosis or misdiagnosis (30). Among patients with colocolic intussusception in our study, the proportion with the above classic triad was as high as 36.4%, but 34.1% were still misdiagnosed. The reason was mainly related to the less severe symptoms of the patients at the initial visit, lack of imaging examination or successive occurrence of the above symptoms (5, 6). Our study also confirmed that, unlike patients with ileocolic intussusception who almost always had abdominal masses, a significantly lower proportion of patients with colonic intussusception had abdominal masses, most of which were located in the left abdomen; instead, patients with colonic intussusception had a significantly higher incidence of rectal masses or prolapse. Both ultrasound and computed tomography are useful tools to diagnose intussusception (31). Ultrasound is recommended first because of its high diagnostic sensitivity (82–95%) in the detection of intussusception and lack of radiation exposure for patients, which was also confirmed by our study (27, 32). Therefore, it should be used at the initial visit for all children with the above symptoms and a clinical suspicion of intussusception, including colocolic intussusception (32).

Given the pathologic lead points found in most colocolic intussusceptions, therapeutic enemas, especially hydrostatic barium enemas, were previously considered to be avoided, and these patients often required open surgery (33). However, treating colocolic intussusception caused by a pathologic lead point is currently considered the same as treating those without a pathologic lead point, involving a careful attempt at reduction using a minimally invasive approach (7). Similar to the findings in the recent systematic review of intussusception, our study suggested that for colocolic intussusception, without peritonitis (e.g., diffuse abdominal tenderness), a therapeutic enema could be performed first (34). The success rates of a therapeutic enema to treat colocolic intussusception with or without pathologic lead points in our study were 50.0% (11/22) and 75.0% (3/4), respectively. A colonoscopy can be performed to investigate the colon wall and identify the pathologic lead point, but routine colonoscopy can only be considered in patients with definite pathologic lead points redemonstrated by imaging examinations after successful therapeutic enemas provided that the hospital has the capacity of emergency colonoscopy (6, 7).

As described in most studies in the literature, active intervention is required for colocolic intussusception caused by pathologic lead points because the presence of lead points may impair complete reduction of intussusception and the recurrence rate is still high even after a successful therapeutic enema (6, 10, 33). According to our study, juvenile polyps are the most common lead point in patients with colocolic intussusception, and using a therapeutic enema followed by colonoscopic polypectomy was found to be a feasible intervention to treat these patients. Among the 8 patients with successful therapeutic enemas who underwent colonoscopy subsequently, 7 (7/8, 87.5%) had their polyps successfully removed, and 1 with a sessile polyp found during colonoscopy underwent segmental colonic resection. Additionally, to the best of our knowledge, the current study reported the largest variety of pathologic lead points of colocolic intussusception other than juvenile polyps and found that almost patients with colocolic intussusception caused by other pathologic lead points had received surgical interventions, except for 1 with colocolic intussusception caused by hereditary angioneurotic edema and 1 with hemangioma. The main reasons for the increased use of open surgery may be as follows. First, most pathologic lead points were rare, and clinicians lacked the awareness and treatment experience of these intussusception types. Second, even if the intussusception was successfully reduced by therapeutic enema, some pathologic lead points, such as familial adenomatous polyposis, synovial sarcoma and lipoblastoma, could not be removed by colonoscopy. Third, after successful reduction by therapeutic enema, some pathologic lead points located outside the colon could not be observed by colonoscopy, such as ileal invagination of the sigmoid colon (12, 23, 24). Abrahams et al. also reported 1 patient with colocolic intussusception caused by a juvenile polyp who had undergone laparoscopic exploration after a successful therapeutic enema, but no abnormality was found; the patient subsequently underwent open surgery because of the recurrence of symptoms (10). Thus, open surgery remains the primary treatment in patients with colocolic intussusception and may be preferable to laparoscopic surgery. Laparoscopy has been widely promoted, but it is still not commonly used in intussusception surgery and is mainly used for exploration. The main reason may be that children with intussusception are mainly hospitalized as an emergency, and laparoscopic surgery cannot guarantee the safety of surgery, especially when intestinal anastomosis is needed. Additionally, the lead point after a successful therapeutic enema is difficult to detect by laparoscopy. However, the identification rate of pathological lead points by open surgery also did not reach 100.0%. For example, the case of colocolic intussusception caused by capillary hemangioma reported by Utsumi et al. could only be accurately resolved by open surgery with colonoscopic assistance (17). Further studies with larger samples are needed to confirm this finding.

**Figure 3:**
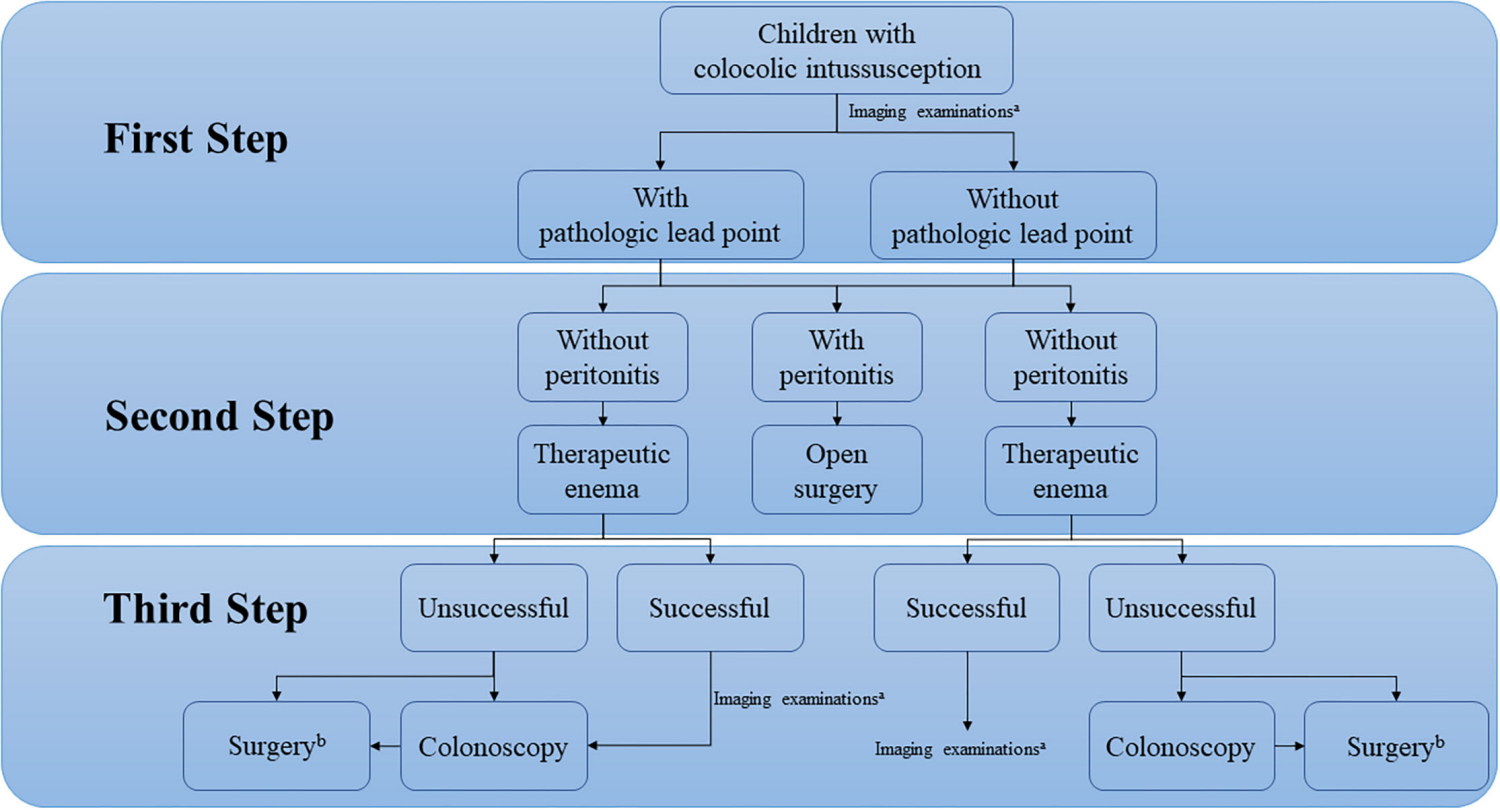
Recommended algorithm for the management of colocolic intussusception in children. ^a^Ultrasound is preferred, combined with other imaging examinations when necessary. ^b^Laparoscopy can be used for exploration and open surgery is recommended as the primary surgical procedure.

With advances in ultrasound and computed tomography, an increasing number of patients can be diagnosed with or without pathologic lead points (17, 33). If the colocolic intussusception caused by pathologic lead points was reduced by therapeutic enema, patients could be hospitalized for a period of time to relieve bowel edema and receive adequate bowel preparation before deciding the best way to manage the pathologic lead points (7). According to our experience in the diagnosis and treatment of colocolic intussusception and a literature review, the recommended algorithm for the management of colocolic intussusception in children is shown in Figure 3. However, except for juvenile polyps, identifying the specific type of lead points preoperatively remains challenging. No study has reported the differences in imaging findings between different pathologic lead points, which would be helpful to develop a more detailed diagnosis and treatment procedure for colocolic intussusception in the future. In addition, as shown in our study, the primary disease of patients with multiple polyps was complicated, and our recommended algorithm cannot be well applied when these patients present colocolic intussusception. They may require individualized treatment due to their rarity.

The study has several limitations: (1) all the included studies were retrospective, and the patient population was small; (2) patients treated successfully by air enema at an outpatient clinic were not included; (3) some studies had not fully reported the results, which may have confounded the findings; and (4) literature reviews did not include all published studies (before 2000), leading to potential bias. A multicenter prospective study of colocolic intussusception is recommended in the future.

In conclusion, a therapeutic enema followed by colonoscopic polypectomy is feasible as a treatment for colocolic intussusception caused by juvenile polyps unless the patient has signs of peritonitis due to bowel perforation; However, surgery is sometimes needed. For patients with colocolic intussusception caused by other pathologic lead points, colonoscopy can be used as a diagnostic method, and open surgery remains the primary treatment. The role of laparoscopic surgery in colocolic intussusception is surgical exploration.

## Data Availability Statement

The original contributions presented in the study are included in the article/supplementary material, further inquiries can be directed to the corresponding author/s.

## Ethics Statement

The studies involving human participants were reviewed and approved by Medical Ethics Committee of Beijing Children's Hospital, Capital Medical University. Written informed consent from the participants' legal guardian/next of kin was not required to participate in this study in accordance with the national legislation and the institutional requirements.

## Author Contributions

JY and YC: study conception and design. JY: data collection. JY, QS, CP, and WP: data analysis and interpretation. JY, QS, CP, WP, and YC: drafting of the manuscript and critical revision. All authors contributed to the article and approved the submitted version.

## Conflict of Interest

The authors declare that the research was conducted in the absence of any commercial or financial relationships that could be construed as a potential conflict of interest.

## Publisher's Note

All claims expressed in this article are solely those of the authors and do not necessarily represent those of their affiliated organizations, or those of the publisher, the editors and the reviewers. Any product that may be evaluated in this article, or claim that may be made by its manufacturer, is not guaranteed or endorsed by the publisher.
